# Artificial Intelligence in Thyroid Cytopathology: Diagnostic and Technical Insights

**DOI:** 10.3390/cancers17213525

**Published:** 2025-10-31

**Authors:** Mariachiara Negrelli, Chiara Frascarelli, Fausto Maffini, Elisa Mangione, Clementina Di Tonno, Mariano Lombardi, Francesca Maria Porta, Mario Urso, Vincenzo L’Imperio, Fabio Pagni, Claudio Bellevicine, Mariantonia Nacchio, Umberto Malapelle, Giancarlo Troncone, Antonio Marra, Giuseppe Curigliano, Konstantinos Venetis, Elena Guerini-Rocco, Nicola Fusco

**Affiliations:** 1 Division of Pathology, European Institute of Oncology IRCCS, 20139 Milan, Italy; mariachiara.negrelli@ieo.it (M.N.); chiara.frascarelli@ieo.it (C.F.); fausto.maffini@ieo.it (F.M.); elisa.mangione@ieo.it (E.M.); clementina.ditonno@ieo.it (C.D.T.); mariano.lombardi@ieo.it (M.L.); francescamaria.porta@ieo.it (F.M.P.); elena.guerinirocco@ieo.it (E.G.-R.); nicola.fusco@ieo.it (N.F.); 2 Department of Oncology and Hemato-Oncology, University of Milan, 20133 Milan, Italy; antonio.marra@ieo.it (A.M.); giuseppe.curigliano@ieo.it (G.C.); 3 Department of Medicine and Surgery, Pathology, IRCCS Fondazione San Gerardo dei Tintori, University of Milano-Bicocca, 20900 Monza, Italy; mario.urso@unimib.it (M.U.); vincenzo.limperio@unimib.it (V.L.); fabio.pagni@unimib.it (F.P.); 4 Department of Public Health, University of Naples Federico II, 80131 Naples, Italy; claudio.bellevicine@unina.it (C.B.); mariantonia.nacchio@unina.it (M.N.); umberto.malapelle@unina.it (U.M.); giancarlo.troncone@unina.it (G.T.); 5 Division of New Drugs and Early Drug Development for Innovative Therapies, European Institute of Oncology IRCCS, Via G. Ripamonti 435, 20141 Milan, Italy

**Keywords:** thyroid cytology, deep learning, artificial intelligence, convolutional neural networks, multiple instance learning, Bethesda system, molecular prediction, explainable AI, multimodal models

## Abstract

**Simple Summary:**

Thyroid nodules are very common, and fine-needle aspiration cytology is the main test used to decide whether a nodule is benign or not. While this test is reliable in most cases, many samples fall into an “indeterminate” category, often leading to unnecessary operations or delays in treatment. New computer-based methods, known as deep learning, can analyze digital images of thyroid cytology slides and may help reduce this uncertainty. By learning patterns that even experienced specialists may overlook, these systems could support pathologists in making faster and more accurate decisions, especially in difficult cases. In this article, we discuss how deep learning has been applied to thyroid cytology, the technical and practical challenges it faces, and how it could eventually help make thyroid cancer diagnosis more precise, consistent, and accessible worldwide.

**Abstract:**

Fine-needle aspiration cytology (FNAC) is the cornerstone of thyroid nodule evaluation, standardized by the Bethesda System. However, indeterminate categories (Bethesda III–IV) remain a major challenge, often leading to unnecessary surgery or delayed molecular testing. Deep learning (DL) has recently emerged as a promising adjunct in thyroid cytopathology, with applications spanning triage support, Bethesda category classification, and integration with molecular data. Yet, routine adoption is limited by preanalytical variability (staining, slide preparation, Z-stack acquisition, scanner heterogeneity), annotation bias, and domain shift, which reduce generalizability across centers. Most studies remain retrospective and single-institution, with limited external validation. This article provides a technical overview of DL in thyroid cytology, emphasizing preanalytical sources of variability, architectural choices, and potential clinical applications. We argue that standardized datasets, multicenter prospective trials, and robust explainability frameworks are essential prerequisites for safe clinical deployment. Looking forward, DL systems are most likely to enter practice as diagnostic co-pilots, Bethesda classifiers, and multimodal risk-stratification tools. With rigorous validation and ethical oversight, these technologies may augment cytopathologists, reduce interobserver variability, and help transform thyroid cytology into a more standardized and data-driven discipline.

## 1. Introduction

Thyroid nodules are common clinical findings, palpable in ~5% of adults but detectable by ultrasound in up to 60% of the population [1]. Fine-needle aspiration cytology (FNAC) according to The Bethesda System for Reporting Thyroid Cytopathology (TBSRTC) is the gold standard for initial risk stratification [2]. However, conventional FNAC is limited by interobserver variability and diagnostic uncertainty, particularly in indeterminate categories (Bethesda III/IV) [3]. These cases frequently result in unnecessary surgery, with benign histology confirmed in 30–40% of resected nodules [4,5]. Molecular testing is a reliable integrative test in indeterminate cases, but the high cost and limited availability restrict its widespread use [6,7,8].

Traditional computer-aided diagnosis (CAD) methods, based on handcrafted features or shallow machine learning, have achieved only limited generalizability, mainly because cytological smears show high intra- and inter-slide variability in staining, fixation, and cell distribution. Deep learning (DL), by contrast, offers an end-to-end approach capable of automatically learning hierarchical morphological patterns directly from digitized slides, without the need for predefined features. These models have shown remarkable success in histopathology and radiology, suggesting that cytology—particularly thyroid FNAC—could be the next frontier for AI-assisted diagnosis [9]. Despite this promise, DL applications in thyroid cytopathology remain relatively underexplored compared to other fields [10]. Existing reviews have primarily offered general overviews of AI in thyroid disease or radiology, with limited focus on cytology-specific challenges and pre-analytical variability [11,12].

In contrast, the present review provides a comprehensive and technically oriented synthesis of deep learning applications in thyroid cytology, connecting the algorithmic principles—ranging from convolutional neural networks to weakly supervised and hybrid frameworks—with their diagnostic implications. Particular attention is given to how pre-analytical factors influence model robustness, how explainability tools can enhance clinical trust, and how these systems might be realistically integrated into routine diagnostic workflows as assistive “co-pilots” rather than replacements for cytopathologists. By focusing on this intersection between technical design and diagnostic feasibility, the review moves beyond descriptive enumeration to offer a framework for critical evaluation and translational readiness of DL systems in thyroid cytology.

## 2. Preanalytical Considerations

Preanalytical variables play a critical role in digital cytopathology, as factors related to staining, specimen preparation, and image acquisition can significantly affect the performance and reliability of downstream computational analyses.

### 2.1. Staining Quality

Staining variability represents one of the most critical and underappreciated sources of pre-analytical heterogeneity in thyroid cytology. Different cytological preparations, such as Diff-Quik, Papanicolaou, and hematoxylin–eosin (H&E) in cell blocks, produce markedly distinct chromatic, textural, and contrast profiles. Each stain provides complementary diagnostic information: Diff-Quik facilitating assessment of overall cellularity and cytoplasmic detail [13], Papanicolaou enabling fine evaluation of nuclear morphology and chromatin [14], and H&E reproducing a histology-like appearance useful for cyto-histologic correlation [15]. This variety poses a formidable challenge for both digital acquisition and downstream DL analysis. Even within the same staining protocol, variations in fixation time, dye concentration, reagent pH, incubation duration, rinsing procedures, and batch-to-batch reagent differences may result in substantial alterations of hue, saturation, and contrast. When digitized, these inconsistencies manifest as domain shifts in color space, whereby nuclear and cytoplasmic tones, edge definition, and background hue differ across slides or institutions. DL models that are not robust to these shifts risk learning color artifacts rather than morphological features truly associated with the underlying pathology.

Classical stain normalization algorithms have been widely employed to mitigate inter-laboratory color variability in digital pathology. Among these, the Macenko and Reinhard methods remain the most established. The Macenko approach operates in the optical density (OD) space, estimating the dominant stain vectors through singular value decomposition (SVD) and reprojecting each image onto a standardized color basis [16]. This technique effectively harmonizes hue and intensity across slides while preserving most morphological information, although it may be sensitive to noise, illumination differences, and overlapping cells [16]. In contrast, the Reinhard method works in the Lab color space, which models human color perception, and aligns the mean and standard deviation of each channel to those of a chosen reference image. It is computationally efficient and performs well when color variations are moderate, but tends to lose accuracy when staining differences or background artifacts are more pronounced [17]. Both techniques provide rapid and accessible color harmonization but rely heavily on the choice of a representative reference image and do not fully address structural or staining artifacts.

Recent deep learning–based normalization models, such as StainNet and Colour Adaptive GAN (CAGAN), have been introduced to overcome the intrinsic limitations of classical algorithms. Unlike Macenko and Reinhard, which rely on global color statistics or linear stain decomposition, these networks learn a non-linear mapping between staining domains directly from data. StainNet employs a compact pixel-wise architecture trained through knowledge distillation, achieving faster inference and improved preservation of fine nuclear detail compared to GAN-based or statistical methods [18]. CAGAN further enhances adaptability by decoupling morphological structure from color attributes, allowing the model to modify chromatic style while maintaining cytological architecture intact. In practice, these approaches better accommodate complex inter-laboratory differences, such as shifts in hue, illumination, or reagent chemistry, resulting in higher consistency across multicenter datasets. However, they remain computationally demanding and require extensive validation to ensure that color transformation does not inadvertently alter diagnostically relevant features [19]. In summary, managing stain variability is essential for developing reliable deep learning models in thyroid cytology. While normalization methods can improve visual consistency, true reproducibility ultimately depends on combining standardized laboratory protocols with robust computational harmonization—an ambitious but achievable goal only through coordinated multicenter efforts.

### 2.2. Specimen Preparation and Slide Digitalization

Cytological preparations for thyroid fine-needle aspiration (FNA) are intrinsically heterogeneous, characterized by variable thickness, irregular topography, and the coexistence of follicular clusters, colloid material, and background debris. These features make both digital acquisition and downstream deep learning (DL) analysis considerably more complex than in histological sections. In conventional smears, thick colloid pools and overlapping follicular groups frequently create abrupt changes in optical density, affecting focus and illumination within the same field. Such variations can distort nuclear and cytoplasmic features, particularly in hyperplastic or cystic nodules, where refractile colloid or air-drying artifacts alter transparency and color balance [20]. Preparation method further affects digital quality. Conventional smears preserve fine cytoplasmic detail and colloid texture but display marked variability in cell overlap and background; liquid-based cytology (LBC), in contrast, produces thinner, cleaner layers that enhance overall uniformity but often disperse fragile follicular and Hürthle cells and may reduce colloid visibility, elements that are morphologically informative for DL models. Moreover, LBC monolayers are not perfectly planar: subtle height variations along the z-axis, especially in poorly cellular samples, make it difficult to maintain uniform focus. This phenomenon, observed in both thyroid and cervical cytology, can result in globally sharp but locally blurred images where diagnostically relevant cells lie slightly out of plane [21,22]. The digitization process itself introduces additional variability. Most whole-slide scanners are optimized for histologic sections and cannot consistently capture the depth of thick thyroid smears. Z-stack scanning mitigates this issue by acquiring multiple focal planes, which is particularly useful for visualizing papillary carcinoma features such as nuclear grooves and pseudoinclusions. Studies in urinary and thyroid cytology have demonstrated that multi-plane imaging improves the detection of nuclear details and interobserver concordance. However, Z-stacking increases acquisition time, file size, and computational load, so most laboratories still rely on single-plane imaging, balancing throughput and optical fidelity [23]. Additional sources of variability, such as scanner type, image compression format (e.g., SVS, TIFF, JPEG2000), and magnification level (20× vs. 40×), can also affect downstream model performance [24,25,26]. From a practical standpoint, it is advisable to employ scanner profiles tailored to the specific preparation type. For conventional thyroid smears, broader focal ranges help capture overlapping follicular aggregates and colloid-rich regions, whereas in LBC slides, narrower z-ranges centered within the circular deposition area yield sharper and more reproducible results. Multi-point focusing within this region, combined with metadata-based quality control, can substantially improve digital consistency and DL inference. The establishment of such preparation-specific scanning profiles represents a feasible and cost-effective approach to enhance reproducibility in digital thyroid cytology.

### 2.3. Human Variability in Region of Interest (ROI) Annotation

Annotation of regions of interest (ROIs) is one of the most significant sources of variability in supervised DL pipelines for thyroid cytology. Unlike histology, where lesions are often well defined, cytological material is inherently heterogeneous: diagnostic cells are scattered among debris, colloid, and non-diagnostic areas. Consequently, even experienced cytopathologists may disagree on which fields truly represent the diagnostic component. These subjective differences, whether in selecting representative follicles, atypical nuclei, or excluding artifacts, can substantially influence model performance and generalizability [27]. Weakly supervised and multiple instance learning (MIL) approaches have been introduced to mitigate these challenges by associating slide-level rather than pixel-level labels. This strategy allows models to learn from whole-slide or case-level diagnoses (e.g., Bethesda categories) without requiring exhaustive manual annotation, reducing dependence on human demarcation. However, these methods remain susceptible to dataset-level bias, including case selection and class imbalance [28,29]. From a practical perspective, improving annotation reliability requires shared standards that clearly define what constitutes a diagnostic region in thyroid cytology. Consensus labeling by multiple experts and the development of public, quality-controlled datasets can mitigate subjectivity and support reproducibility across studies. Moving forward, hybrid annotation workflows, in which AI pre-selects candidate regions for expert review through pre-segmentation or attention maps, may offer a balanced compromise between accuracy and scalability, turning annotation into a collaborative rather than purely manual task [30].

### 2.4. Data Quality, Inclusion Criteria, and Domain Shift

The diagnostic landscape of thyroid cytology poses unique challenges for dataset construction and quality control. Across published studies, data inclusion criteria vary substantially—some focus exclusively on unequivocal benign (Bethesda II) and malignant (Bethesda VI) cases, while others also incorporate indeterminate or non-diagnostic categories (Bethesda I, III, and IV). Although this selective inclusion simplifies model training and improves apparent accuracy, it limits the algorithm’s ability to manage real-world diagnostic uncertainty. In clinical practice, it is precisely the indeterminate categories that generate the greatest need for diagnostic support; excluding them therefore undermines the translational value of such models [31]. A second limitation lies in the handling of suboptimal or low-cellularity slides. Many datasets exclude FNA smears with poor fixation, thick colloid, or extensive blood contamination, conditions frequently encountered in routine practice. This “data cleaning” artificially inflates performance metrics and may produce models that perform well on idealized slides but fail on typical daily cases. In thyroid cytology, where cellular adequacy and background composition vary widely between centers, maintaining representative heterogeneity is essential for external validity [32]. Another critical issue is domain shift, the systematic discrepancy between training and testing data caused by differences in staining, scanning equipment, preparation method, or patient population. A DL model trained on LBC slides from a single institution may underperform when applied to conventional smears or slides digitized at different resolutions. In thyroid FNA, where both preparation types coexist, this risk is particularly high. Domain adaptation and color normalization can partly mitigate the problem, but they cannot replace a diverse and representative dataset [33,34]. In conclusion, robust dataset design should balance data quality with sufficient heterogeneity to reflect the variability inherent in routine thyroid cytology, thereby supporting the development of DL models that generalize across institutions and preparation types.

## 3. Architectural Variables

The optimal deep learning (DL) model for thyroid fine-needle aspiration cytology (FNAC) must address both technical variability and the biological diversity of lesions. Traditional machine learning (ML) approaches based on handcrafted features—such as nuclear texture or geometric descriptors—showed limited reproducibility across centers, as cytological smears display high intra-slide variability. In contrast, DL architectures autonomously learn multilevel representations from raw image data, enabling them to identify non-linear and context-dependent morphological cues that are often difficult to quantify by eye. This property makes DL particularly suitable for thyroid cytology, where diagnostic features such as nuclear grooves, chromatin clearing, or colloid background may appear focal and heterogeneous [35,36,37,38].

### 3.1. Convolutional Neural Networks (CNNs)

CNNs remain the foundation of most DL pipelines in cytopathology. By applying trainable filters across the image, they extract increasingly abstract spatial features—from edges and textures to complex cell arrangements—without predefined feature engineering [39,40]. Classical architectures combine convolutional and pooling layers with non-linear activations (e.g., ReLU) and fully connected layers for classification [41,42]. In thyroid cytology, CNN-based models have shown particular promise in distinguishing benign from malignant lesions by leveraging subtle morphological cues. Lin et al. demonstrated that a CNN-based screening approach applied to thyroid cytology WSIs could accurately differentiate benign colloid-rich nodules from papillary and microfollicular carcinomas, capturing fine variations in nuclear morphology and colloid density [43]. Recently, CNN models have evolved from early architectures such as VGGNet and ResNet to more efficient designs like EfficientNet, which offer a good balance between accuracy and computational cost. When cytology datasets are small, transfer learning—using models pre-trained on large image collections such as ImageNet—can improve stability and speed up training [44,45,46,47]. Despite these advantages, CNNs still require large, well-curated datasets and often act as “black boxes”. Visualization tools such as Grad-CAM or SHAP can help confirm that model predictions are based on true diagnostic areas rather than color or scanning artifacts [11].

### 3.2. Multiple Instance Learning (MIL)

MIL is a specific weakly supervised framework particularly suited for WSI classification in pathology and cytology [48]. In this setting, each slide is treated as a ‘bag’ of smaller instances (patches or tiles) [49]. Labels are assigned only at the bag level, not to individual instances: this is particularly useful in cytology, where manual annotations are time-consuming and subject to variability [50]. Classical MIL assumes a slide is positive if at least one patch is positive, but modern implementations employ attention-based pooling to learn which patches contribute most to the final prediction, improving both performance and interpretability [51]. This strategy mirrors the reasoning process of cytopathologists, who evaluate the smear integrating focal atypia, background, and colloid distribution rather than analyzing isolated cells. Recent implementations have adopted attention-based pooling, allowing the network to assign greater importance to diagnostically relevant regions and to generate interpretable heatmaps. In a study published in 2025, by combining a custom CNN backbone (TCS-CNN) with attention-based MIL to classify WSIs into Bethesda II, IV, and VI categories, a 97% accuracy was reached without pixel-level annotation [52]. MIL represents an efficient alternative to fully supervised models but remains influenced by dataset imbalance and sampling bias. When indeterminate or low-cellularity cases are underrepresented, the model may overfit to clear-cut examples, limiting generalizability. Ensuring adequate representation of all Bethesda categories and preparation types is therefore crucial for future studies [53].

### 3.3. Hybrid DL Platforms

Hybrid strategies combining supervised and weakly supervised signals may offer the best compromise for complex domains like thyroid cytology. In 2021, a two-stage refined CNN demonstrated accurate benign-versus-malignant classification of thyroid FNAC slides by using expert-selected cellular regions to guide the model, improving robustness and reducing misclassification. A subsequent 2023 study applied a semi-supervised Noisy Student approach to cervical cytology and achieved performance comparable to fully supervised models, highlighting the potential of this method for datasets with limited annotations [54,55]. In thyroid cytology, such hybrid and semi-supervised strategies are especially promising for indeterminate categories (Bethesda III–IV), where explicit region-level annotations are scarce but slide-level diagnoses are available. By generating both diagnostic probabilities and attention maps, these systems provide interpretable outputs that can assist cytopathologists in verifying predictions and identifying diagnostically relevant areas, supporting the integration of AI into real-world workflows [52,56,57,58,59,60,61].

## 4. Toward a Reliable Digital Cytodiagnostic Pipeline

**Figure 1:**
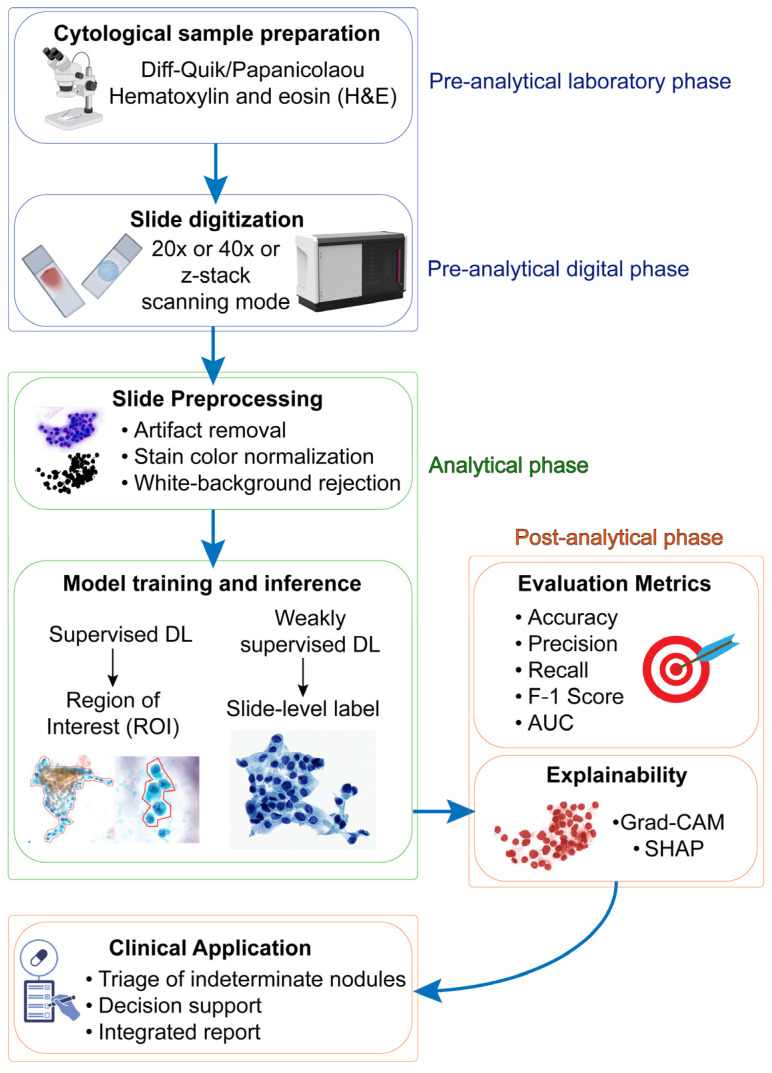
Deep learning workflow in thyroid cytology. The diagram outlines the end-to-end pipeline across four phases. Pre-analytical (laboratory): cytological sample preparation with common stains (Diff-Quik, Papanicolaou, H&E). Pre-analytical (digital): slide digitization at 20×/40× magnification, with or without Z-stack acquisition (improved focal coverage vs increased time, file size, and computational load). Analytical: preprocessing (artifact removal, color normalization, white-background rejection), followed by model training/inference using supervised (ROI-based) or weakly supervised (slide-level/MIL/attention) strategies. Evaluation and post-analytical: performance assessment with standard metrics (accuracy, precision, recall, F1-score, AUC) and integration of explainability tools (e.g., Grad-CAM, SHAP) to support interpretability. Clinical outputs: triage of indeterminate nodules, diagnostic decision support (e.g., Bethesda categorization), and integrated reporting. Detailed assumptions and trade-offs for each block are discussed in Section 2, Section 3 and Section 4.

Although no DL system is yet approved for routine use in thyroid cytology, several applications have been proposed. These include triage systems that flag low-risk slides for deferred review, decision-support tools that suggest Bethesda categories with confidence scores, and telepathology platforms [23,59,62,63]. All these together can theoretically be integrated into a clinical diagnostic workflow, as portrayed in Figure 1.

### 4.1. Co-Pilot

The most immediate application of DL models in thyroid FNAC is as diagnostic support tools, often described as co-pilots [58,64]. Instead of replacing the cytopathologist, these systems can assist in routine thyroid workflows by prioritizing cases, flagging atypical or suspicious smears, and providing second-opinion Bethesda category suggestions, particularly for diagnostically challenging cases (Bethesda III–IV). When embedded into digital pathology viewers (e.g., QuPath or SlideViewer), DL models can deliver real-time overlays and ROI highlights, enabling cytopathologists to correlate AI outputs with key thyroid-specific cytomorphological features such as nuclear grooves, pseudoinclusions, or colloid density [65]. Although several co-pilot DL systems have been developed for thyroid cytology, none are yet validated for clinical use. These tools can prioritize challenging smears, flag atypical areas, or provide Bethesda category suggestions with confidence scores. Their true value lies in assisting, not replacing, the cytopathologist. When integrated into digital viewers, they can overlay attention maps highlighting features such as nuclear grooves or pseudoinclusions. However, their adoption still depends on standardized interfaces, explainable outputs, and demonstration of clinical benefit in workflow-based studies.

### 4.2. Bethesda Classifiers

DL models have been evaluated for automatic Bethesda categorization of thyroid FNAC slides. Most approaches use patch-level inference aggregated at the slide level through attention pooling or ensemble voting. CNN-based pipelines remain the most common, as in ThyroidEffi 1.0, which achieved high performance across Bethesda II, V, and VI (macro F1 = 0.897; AUC up to 0.98) [56]. More recently, transformer-based architectures have been explored: Zhu et al. developed vision–language models capable of generating Bethesda-style reports directly from digital images and textual inputs [66]. Despite these advances, fine-grained classification across all six Bethesda categories remains difficult, largely due to interobserver variability, overlapping cytomorphology, and annotation noise. The greatest challenge lies in indeterminate cases (Bethesda III–IV), which drive clinical uncertainty and often lead to unnecessary surgery or delayed molecular testing. Several studies have investigated whether DL can help refine risk stratification in this setting. For example, Zhong et al. [59] combined ultrasound radiomics with clinical features to classify Bethesda III nodules (AUC = 0.82), while Poursina et al. [67] and a 2025 meta-analysis [68] reported promising accuracy (pooled AUC ≈ 0.85) for AI-based reclassification of indeterminate nodules, though with considerable heterogeneity and limited external validation. Overall, DL-based classifiers show encouraging results, but evidence remains preliminary. Prospective, multi-center studies are essential to establish robustness, and future work should assess whether AI-driven risk stratification can safely guide conservative management in low-risk indeterminate cases.

### 4.3. Molecular Classifiers

The molecular landscape of thyroid tumors has become integral to cytological diagnosis and risk assessment [69,70,71,72,73,74,75]. The fifth edition of the WHO Classification of Thyroid Tumors incorporates key genetic alterations (i.e., BRAF V600E, RAS mutations, RET/PTC rearrangements, and PAX8–PPARG fusions) into the diagnostic framework for follicular-derived neoplasms [76]. With the growing use of NGS panels, cytopathologists now routinely integrate molecular data into FNAC interpretation. Commercial tools such as ThyroSeq, Afirma GSC, and ThyGeNEXT/ThyraMIR are widely applied in Bethesda III–IV nodules, providing genomic risk profiles that guide management [77,78]. In parallel, DL models have been explored for their ability to predict molecular alterations directly from cytological images. Early studies suggest that CNNs can capture genotype-associated morphologic features, particularly in BRAF and RAS-mutated tumors [79]. While still experimental, such models could eventually be integrated into multimodal pipelines that combine cytology, molecular, and clinical data to improve risk stratification. In the long term, the integration of cytological, molecular, and clinical data through multimodal DL frameworks could help move thyroid cytology toward a precision-medicine model. Such tools are not expected to replace current molecular assays but to complement them—linking morphology, genotype, and outcome in a unified predictive continuum [80].

## 5. Conclusions and Future Directions

DL has emerged as a promising tool for improving diagnostic accuracy and workflow efficiency in thyroid cytology. Nevertheless, its translation from proof-of-concept to clinical reality remains constrained by technical, methodological, and organizational factors. Most published studies rely on relatively small, single-center datasets collected under heterogeneous staining, fixation, and digitization conditions: this lack of standardization limits external generalizability and hinders regulatory approval. In addition, variable annotation quality, inconsistent Bethesda categorization, and exclusion of low-cellularity or suboptimal smears often result in overoptimistic performance metrics that do not reflect real-world complexity.

Beyond quantitative accuracy, reproducibility and explainability are emerging as essential prerequisites for clinical acceptance. Models must demonstrate robustness to stain variability, scanner configuration, and preparation type, ensuring consistent behavior across institutions. While visualization methods such as Grad-CAM and SHAP have increased transparency, they remain qualitative in nature. This is particularly critical in indeterminate categories (Bethesda III–IV), where uncertainty has the greatest impact on patient management.

Legal, ethical, and regulatory frameworks will play a decisive role. Data privacy, algorithmic bias, and liability remain unresolved, and future deployment must balance innovation with accountability.

Looking ahead, DL applications most likely to reach the clinic include Bethesda classifiers, triage support, and multimodal models integrating cytology with molecular and clinical data. These systems should be evaluated not only by accuracy, but also by their ability to improve outcomes, reduce interobserver variability, and optimize resource use. With collaborative validation, ethical oversight, and a focus on clinical utility, AI can evolve from experimental prototypes into reliable co-pilots—supporting cytopathologists and transforming thyroid cytology into a more standardized, data-driven discipline.

## Data Availability

Data sharing is not applicable.
